# Greater mechanical temporal summation of pain in Latinx-Americans and the role of adverse life experiences

**DOI:** 10.1097/PR9.0000000000000842

**Published:** 2020-09-01

**Authors:** Fenan S. Rassu, Jessica C. Luedke, Namrata Nanavaty, Vani A. Mathur, Mary W. Meagher

**Affiliations:** a Department of Psychological and Brain Sciences, Texas A&M University, College Station, TX, USA; b Department of Psychology, University of South Carolina, Columbia, SC, USA; c Texas A&M Institute for Neuroscience, Texas A&M University, College Station, TX, USA

**Keywords:** Temporal summation, Ethnic differences, Latinx, Ethnic discrimination, Adverse life experiences, Pain

## Abstract

**Introduction:**

Adverse life experiences disproportionately impact Latinx-Americans and are related to greater chronic pain rates. However, little is known about how adversities interact with central pain mechanisms for the development of later pain among Latinx-Americans.

**Objectives:**

The current study examined the relationship between adverse life experiences (eg, trauma and ethnic discrimination) and correlates (eg, social status) with mechanical temporal summation of pain (a proxy measure of central sensitization) between pain-free U.S. native Latinx (n = 65) and non-Hispanic White (NHW) (n = 51) adults.

**Methods:**

Participants completed self-report adverse life experience and correlational measures regarding childhood and adulthood and a mechanical temporal summation protocol.

**Results:**

Relative to NHWs, Latinx-Americans reported experiencing significantly greater trauma, discrimination, and lower social status during childhood and adulthood, along with greater temporal summation. Contrary to hypotheses, recent and lifetime experiences of ethnic discrimination significantly correlated with less temporal summation among Latinx-Americans. Decreases in objective and subjective social status across the lifespan (childhood to present day) correlated with greater temporal summation for Latinx-Americans. However, r-to-z transformation analyses confirmed that significant adversity and social status correlations observed among the Latinx group did not significantly differ from NHW participants.

**Conclusions:**

The present findings highlight the complex association between adverse experiences, adverse experience risk factors, and pain for Latinx-Americans. Given the disproportion of experienced pain and adversity among Latinx-Americans, the current findings suggest that a better understanding of the unique adversities for this sample may help elucidate the mechanisms underlying the relationship between adversities, adversity correlates, and pain risk for Latinx-Americans.

## Abstract 2

Supplemental Digital Content is Available in the Text.

Latinx-Americans showed greater temporal summation, adversity, and adversity correlates relative to non-Hispanic Whites. Discrimination and lifespan social status change inversely related to summation for Latinx-Americans.

## 1. Introduction

Latinx-Americans—Americans of Latin-American origin or descent—disproportionately experience disparities for many health conditions,^16,37,59^ including pain.^10,15,17,29,31,35,58,62,68^ Disrupted endogenous pain mechanisms, such as enhanced central sensitization, underlie greater risk for chronic pain. Central sensitization is a phenomenon characterized by an amplification of neural signaling within the central nervous system that elicits pain hypersensitivity.^5,7,64,65,77,84,88,89^ A noninvasive proxy measure of central sensitization in humans is temporal summation of pain, a progressive increase in pain intensity ratings to repetitive noxious stimuli.^27^ Temporal summation of pain is heightened in individuals with chronic pain,^65,78^ providing a potential mechanism underlying the risk for clinical pain development and persistence. Other people of color, such as African Americans with clinical pain (ie, acute and chronic pain) show enhanced temporal summation relative to non-Hispanic White (NHW) counterparts.^20,38,70^‬‬‬‬‬‬‬‬‬‬‬‬‬‬‬‬‬‬‬‬‬‬‬‬‬‬‬‬‬‬‬‬ ‬‬‬‬‬‬‬‬‬‬‬‬‬‬‬‬‬ Pain-free African Americans show enhanced summation relative to NHWs,^13,55^ suggesting that the risk for greater pain severity may manifest before clinical pain development. If heightened temporal summation occurs in the current study among pain-free Latinx-Americans relative to NHWs, this may suggest that amplified central pain processing could be crucial to explaining the greater chronic pain severity seen in Latinx-Americans with clinical pain relative to NHWs.^10,15,31,35,58,62,68^ However, the psychosocial factors that may act on endogenous pain summation mechanisms to contribute to pain disparities are relatively unknown.‬‬‬‬‬‬‬‬‬‬‬‬‬‬‬‬‬‬‬‬‬‬‬‬‬‬‬‬‬‬‬‬‬‬‬‬‬‬‬‬‬‬‬‬‬‬‬‬‬‬‬‬‬‬‬‬‬‬‬‬‬‬‬

One psychosocial factor that may drive enhanced central sensitization is exposure to adverse life experiences. Many studies find that exposure to traumatic events (eg, physical, sexual, and emotional)—an adverse life experience—are related to pain development^9,42,43,74,79,82^ and exacerbation.^3,90–93^ Moreover, studies find greater adversity and trauma positively predict enhanced central sensitization markers.^90,91,93^ Considering Latinx-Americans disproportionately experience childhood trauma relative to NHWs,^50,72^ traumatic experiences may relate not only with the greater pain observed among Latinx-Americans,^10,15,17,29,31,35,58,62,68^ but also with enhanced central sensitization markers that underlie greater risk for chronic pain.

Latinx-Americans also experience unique, pervasive adverse life experiences that may relate with pain disparities, such as racial/ethnic discrimination.^14,39,86^ Ethnic discrimination among Latinx-Americans in the United States is a common phenomenon: 62% of U.S.-born Latinx-Americans report experiencing discrimination or being mistreated because of their ethnicity.^47^ Latinx-Americans not only experience discrimination more than NHWs, they likewise appraise it as more stressful.^49^ Studies show that perceived racial/ethnic discrimination predicts greater clinical pain severity and laboratory pain sensitivity for African Americans.^14,25,54,86^ Perceived racial discrimination likewise predicts greater laboratory pain sensitivity for African Americans but not NHWs.^39^ Moreover, 2 recent studies find that discrimination predicts greater clinical pain severity among Latinx-Americans.^6,23^ Taken together, these studies implicate perceived racial/ethnic discrimination as a risk factor for greater central sensitization and clinical pain experiences in minoritized people of color. However, the relationship between ethnic discrimination and temporal summation of pain in Latinx-Americans remains unexplored.

A correlate of adverse life experiences related to Latinx-American pain disparities that likewise warrants attention is social status. Social status is the relative rank an individual holds in a social hierarchy. Low social status relates to poorer physical health,^1,2,21,94^ including for Latinx-Americans.^34,36,73^ For pain, objective^8,28,30,32,40,44,48,57,62,81^ and subjective social status markers^87^ relate with greater chronic pain rates and severity, along with worse pain outcomes.^8,28,30,44,57,62,81^ Poverty status likewise inversely correlates with temporal summation among middle-to-older aged adults with knee osteoarthritis.^38^ Although an earlier study found that the Latinx-American subgroup with the lowest social status was at the greatest risk for reporting chronic abdominal pain,^53^ the relationship between social status and temporal summation of pain in Latinx-Americans is unknown. The relationship between social status and temporal summation may help explain clinical pain disparities for demographic groups who disproportionately fall into lower socioeconomic strata, such as Latinx-Americans.^33,62^

This study therefore examined ethnic differences in temporal summation among pain-free Latinx-American and NHW Americans. The study also examined the relationship between adverse experiences and temporal summation between these 2 groups to examine whether adverse experiences correlate with temporal pain summation. We predicted Latinx-Americans would report greater levels of trauma and ethnic discrimination along with lower social status relative to NHWs. Moreover, we also hypothesized that Latinx-Americans would show heightened temporal summation compared to NHWs. Finally, we hypothesized that trauma, discrimination, and social status would correlate with temporal summation across ethnicities, but particularly among Latinx-Americans.

## 2. Methods

The institutional review board at Texas A&M University approved this study. All participants provided informed consent and participated between January 2018 and May 2019.

### 2.1. Participants

Participants self-reported their ethnicity as either “non-Hispanic White” or “Hispanic/Latino.” To control for nativity/migration status, only participants reporting being born and raised in the United States were invited.^46^ To establish if enhanced sensitization occurs before the clinical pain onset, pain-free undergraduate students between the ages of 18 and 40 without chronic health conditions completed the study. Participants stemmed from a psychology course and received course credit for their participation. To control for other confounds beyond the study's objectives that could affect laboratory pain sensitivity, exclusionary criteria included: (1) current use of any prescription drugs (except for hormonal contraceptives), (2) history of fainting spells, (3) any skin condition/numbness on the hands or forearms, (4) history of neurological disorders, (5) current chronic pain or health condition, and (6) use of allergy or pain medication within 24 hours before the experiment.

### 2.2. Sample size calculation

Based on findings from a meta-analysis examining racial and ethnic differences in experimental pain sensitivity, an a priori power analysis determined the sample size required per ethnicity to reveal significant differences in pain sensitivity.^45^ In estimating with a medium effect (f = 0.25), 80% power, α = 0.05, with 2 groups and 2 pain measurements (ie, average 180 g, average 300 g) for a between-factors effect, the required sample size is 98 total.

### 2.3. General overview of procedures

The current study derived data from a larger study examining Latinx-American laboratory pain and emotion sensitivity comprising 134 participants. However, only 116 participants completed temporal summation procedures due to early study terminations from exclusionary criteria, withdrawal, time constraints, or technical difficulties. The approximately 4-hour parent study occurred during a single session within a temperature-controlled, sound-attenuated room. Participants were prescreened for inclusion/exclusion criteria before being invited to the laboratory, and again on the day of testing. If eligible, participants then filled out several questionnaires to assess background characteristics. After being affixed with psychophysiological leads, participants completed emotional and physical (eg, mechanical and thermal) processing tasks. Each task occurred with at least a 2-minute enforced rest between tasks to reduce carryover effects. Before the Mechanical Temporal Summation Task, participants completed 2 heat sensory tests on the contralateral, nondominant side of their body.

A single experimenter with approximately 8 years' experience administering laboratory pain tests (Rassu) conducted the study. Participants were alone in the experiment room while answering questionnaires to minimize observer effects.

### 2.4. Measures

#### 2.4.1. Trauma

The Early Traumatic Inventory Self-Report (ETISR) assessed traumatic life events before 18 years of age. The ETISR is a 27-item questionnaire that summed responses to assess traumatic life events in 4 domains (general, physical, emotional, and sexual trauma), along with disturbance and dissociative symptoms in response to the most distressing event.^11^

#### 2.4.2. Ethnic discrimination

The General Ethnic Discrimination Scale (GEDS) assessed appraised stress severity (“not at all stressful” to “extremely stressful”) and frequency (“never” to “almost all the time”) of 17 perceived discriminatory events (eg, *How often have you been treated unfairly by strangers because of your race/ethnic group?*) during the past 2 years (recent) and their entire life (lifetime).^49^ Evidence suggests scales modeled the perceived ethnic discrimination construct equally well across racial/ethnic groups, including Latinx-Americans.^49^

#### 2.4.3. Social status

We assessed both objective (ie, parental education level and family household income) and subjective social status. In some instances, subjective social status is a unique^21^ and better^2,76^ health status predictor relative to objective status. The current study classified social status as childhood and present day. The U.S. version of the MacArthur Scale of Subjective Social Status was used to measure subjective social status.^1^ To measure childhood subjective social status, participants recorded their parent's social status during childhood (ie, 0–12 years old) relative to the greater U.S. on an illustrated nine-step ladder in which the top rung represents those with the most education, money, and respected jobs (highest status), whereas the bottom rung of the ladder represents those with the least education, money, and respected jobs (lowest status). Participants likewise recorded their own personal, present day subjective social status using the same measure. Evidence suggests that subjective social status significantly correlates with objective indicators of socioeconomic status such as education history, income, and employment status.^2^ Furthermore, subjective social status ladders have been used in several studies with ethnically diverse participants, including Latinx-Americans.^61,95^

We also calculated change in objective (Δ Objective Social Status = present family household income minus childhood family household income) and subjective social status across the lifespan (Δ Subject Social Status = present personal subjective social status minus childhood parental social status), with more positive values indicating greater increases in social status across the lifespan. Change in social status across the lifespan represents a shift from one level of social status to another within a given social hierarchy.^71^ Comparing participants' social status with their parents is a common method for determining social mobility, or change in social status across the lifespan.^41,48,52,75^

### 2.5. Mechanical temporal summation procedure

**Figure 1.**
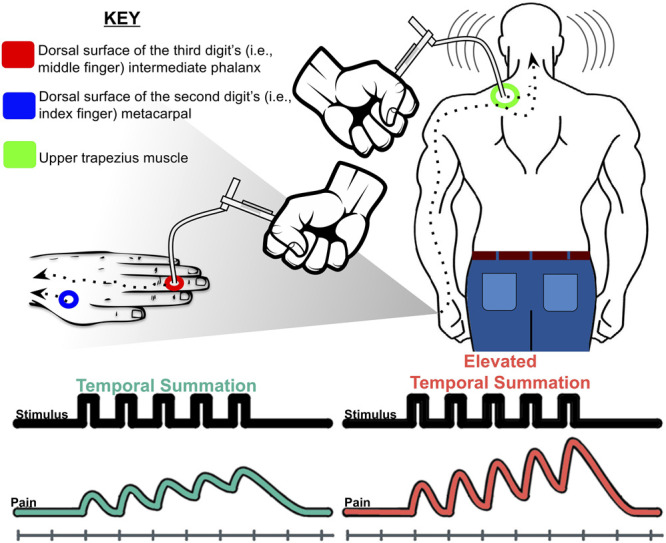
Mechanical temporal summation of pain procedure described in the current study. Participants were first assessed for initial pain after receiving a single contact and verbally rating the intensity of the pain from the single contact on a scale ranging from 0 (“no pain”) to 100 (“the most intense pain imaginable”). Participants then received a series of 10 additional contacts at a rate of one contact per second at the same body site. Upon completing 10 contacts, participants rated the peak or greatest pain intensity experienced during the 10 contacts. This single and 10 contact procedures occurred twice on each anatomical site for both the 180 and 300 g monofilaments. Temporal summation at each site was first calculated by averaging the initial and peak pain responses across the 2 trials at each site then subtracting pain intensity ratings of the single contact from the peak pain intensity.

A Mechanical Temporal Summation Task assessed summation of pain ratings to a presented series of mechanical stimuli (Fig. 1). Temporal summation refers to increased perceived pain from either C- or Aδ-fiber stimulation by repetitive, constant-intensity, noxious stimuli delivered at frequencies greater than 0.33 Hz.^63^ Using 180 and 300 g calibrated nylon monofilaments designed to deliver a consistent gram force upon the filament's bend, participants were assessed on 3 locations across the participants' dominant side: the dorsal surface of the third digit's intermediate phalanx, the dorsal surface of the second digit's metacarpal, and the upper trapezius muscle (for details, see Fig. 1). Given the high intercorrelation across the temporal summation indices, total temporal summation was then calculated by subtracting average initial pain intensity ratings across the 3 sites from average peak pain intensity ratings across the same sites.^12,13,38^ The order of testing across the 3 anatomical sites were randomized per individual. Before pain assessment, participants completed pain rating training until confident with their own ability.

### 2.6. Potential covariates

The current study focused on the relationship between adversities, adversity correlates, and temporal summation of pain. However, negative mood is likely related to adversities, adversity correlates, and pain. To ensure there were no confounding effects, we included sex, depression, and perceived stress as potential covariates. The Center for Epidemiological Studies Depression 20-item assessed depressive symptoms within the previous week.^67^ Higher Center for Epidemiological Studies Depression scores indicate more severe depressive symptomology, with a 20 cutoff score reflecting an adequate tradeoff between sensitivity and specificity for clinical depression risk.^85^ The Perceived Stress Scale 10-item assessed perceived stress within the previous 2 weeks.^19^ Although the Perceived Stress Scale is not a diagnostic instrument, thus absent of cutoff scores, higher scores indicate greater perceived stress. However, controlling for the 3 variables did not change the effect size, direction, or significance of relationships between temporal summation of pain and either the adversity or adversity correlate variables.

### 2.7. Data analysis

When values were missing because of lack of response or equipment malfunction, pairwise deletion was used to exclude participants from those particular analyses.^4^ Differences in continuous variables were examined with *t* or F tests, whereas categorical data used χ^2^ analyses. Significance was set at α < 0.05 (2-tailed). Partial eta squared (${\text{η}}_{p}^{2}$) was the effect size metric for F tests, with 0.009, 0.0588, and 0.1379 corresponding to small, medium, and large effect sizes, respectively.^18,69^ Phi φ and Cramer's V were used as a measures of effect size for χ^2^. Cohen's *d* was the effect size metric for *t* tests.^18^ SPSS 23.0 (IBM, Armonk, NY) was used for all analyses.

#### 2.7.1. Primary analyses

A series of one-way analyses of variance and χ^2^ analyses were used to examine ethnic differences between adversities and adversity correlates. To determine whether temporal summation occurred at each site for the 180 and 300 g filaments, we used paired *t* tests to compare the average pain rating after a single contact to the average maximal pain rating after 10 contacts, collapsed across ethnicity. Then, a two-way ethnicity (between: NHW, Latinx) X total body temporal summation across monofilament weights (within: 180, 300 g) repeated-measures analysis of variance evaluated ethnic differences in temporal summation (average total body peak pain minus average initial total body pain difference scores). Because transformations could not correct normality violations, nonparametric Spearman correlations analyzed the relationships between demographics, self-report measures, and total body average temporal summation of pain, separated by ethnicity groups. We then compared significant correlation coefficients for temporal summation across ethnicities using r-to-z transformations.

## 3. Results

### 3.1. Background characteristics

Table 1 displays adversities and adversity correlate comparisons between Latinx-Americans and NHWs. Participants did not differ in sex, age, depressive symptoms, or perceived stress. Latinx-Americans reported greater frequency and stress appraisal of lifetime and recent ethnic discrimination. Latinx-Americans also reported greater experiences of total trauma and trauma symptoms, along with greater emotional and sexual traumatic experiences. Several participants across ethnicities reported not knowing their current or childhood family income (Table 1). Latinx-Americans reported lower household income at both time periods, along with lower parental education and childhood subjective social status relative to NHWs. There was no ethnic difference in current subjective social status. Finally, Latinx-Americans reported greater increases in subjective social status from childhood to present day relative to NHWs, suggesting subjective social status grew to a greater degree over the lifespan for Latinx-Americans. However, there was no difference for change in objective social status (ie, household family income) from childhood to present day between ethnicity groups.

**Table 1:** Background characteristics by ethnicity.

Continuous	Non-Hispanic White				Latinx-American				F	*P*	${\text{η}}_{\text{p}}^{2}$
	Cronbach α	N	Mean	SD	Cronbach α	N	Mean	SD			
Demographic											
Age		51	19.12	0.13		65	18.86	0.11	2.30	0.132	0.020
Mood											
Depressive symptoms (CES-D; 0–60)	0.87	51	13.98	8.70	0.85	65	15.37	8.23	0.77	0.381	0.007
Perceived stress (PSS; 0–40)	0.85	51	17.96	5.86	0.87	65	18.34	6.75	0.10	0.752	0.001
Trauma frequency (ETISR)											
Total (0–27)*	0.71	51	5.41	3.37	0.83	64	7.19	4.81	4.99	0.027	0.042
General (0–11)	0.57	51	2.33	1.85	0.51	64	2.70	1.89	1.11	0.295	0.010
Physical (0–5)	0.78	51	2.08	1.70	0.77	64	2.23	1.71	0.24	0.626	0.002
Emotional (0–5)*	0.77	51	0.82	1.31	0.85	64	1.58	1.80	6.32	0.013	0.053
Sexual (0–6)*	0.68	51	0.18	0.59	0.82	64	0.67	1.36	5.90	0.017	0.050
Ethnic discrimination (GEDS)											
Lifetime (17–102)‡	0.89	51	22.39	5.75	0.93	65	29.23	11.60	14.84	<0.001	0.115
Recent (17–102)‡	0.83	51	21.41	4.84	0.90	65	26.97	9.64	14.13	<0.001	0.110
Appraisal (17–102)‡	0.91	51	20.57	8.44	0.95	65	32.65	17.87	19.81	<0.001	0.148

Categorical	Non-Hispanic White		Latinx-American		χ^2^	*P*	φ, Cramer's V
	N	Median or %	N	Median or %			
Demographic							
Gender (female)	51	45%	65	63%	3.74	0.053	0.179
Mood							
Depressive symptoms >20 (CES-D; clinical depression)	51	27%	65	32%	0.32	0.572	0.053
Trauma symptoms (ETISR)							
Disturbance (yes)*	51	27%	64	45%	3.87	0.049	0.183
Dissociative (yes)*	51	12%	64	27%	3.88	0.049	0.184
Objective social status							
Current household income (1–9)‡	43	9	60	7	32.61	<0.001	0.563
Childhood household income (1–9)†	41	9	55	7	25.24	0.001	0.513
Father's education (1–9)†	51	7	64	5	27.12	0.001	0.486
Mother's education (1–9)‡	51	7	64	6	31.31	<0.001	0.522
Δ objective social status (−8 to 8)	41	0	52	0	9.46	0.396	0.319
Subjective social status							
Current subjective social status, U.S. (1–9)	51	6	64	5	12.95	0.073	0.336
Childhood subjective social status, U.S. (1–9)‡	51	6	64	4	32.72	<0.001	0.533
Δ subjective social status (−8 to 8)*	51	−1	64	0	19.37	0.022	0.410

Income coded 1 = less than $5,000, 2 = $5,000 through $11,999, 3 = $12,000 through $15,999, 4 = $16,000 through $24,999, 5 = $25,000 through $34,999, 6 = $35,000 through $49,9997 = $50,000 through $74,999, 8 = $75,000 through $99,999, 9 = $100,000 and greater; Parent's Education Coded 1 = elementary school or less, 2 = middle school, 3 = some high school, 4 = high school graduate/GED equivalent, 5 = postsecondary school other than college, 6 = some college, 7 = college graduate, 8 = some graduate school, 9 = graduate degree.

* *P* < 0.05.

† *P* < 0.01.

‡ *P* < 0.001.

CES-D, The Center for Epidemiological Studies-Depression; ETISR, Early Traumatic Inventory Self-Report; GEDS, General Ethnic Discrimination Scale; PSS, Perceived Stress Scale.

### 3.2. Temporal summation phenomenon

Collapsed across ethnicity groups, average pain intensity ratings after the 10th contact was significantly greater than average pain intensity ratings after the first contact at the phalanx, metacarpal, and trapezius for both the 180 and 300 g von Frey monofilaments (*P*'s < 0.001, *Cohen's* d's ≥ 1.085), indicating temporal summation across sites and stimulus intensities.

### 3.3. Ethnic differences in temporal summation

**Figure 2.**
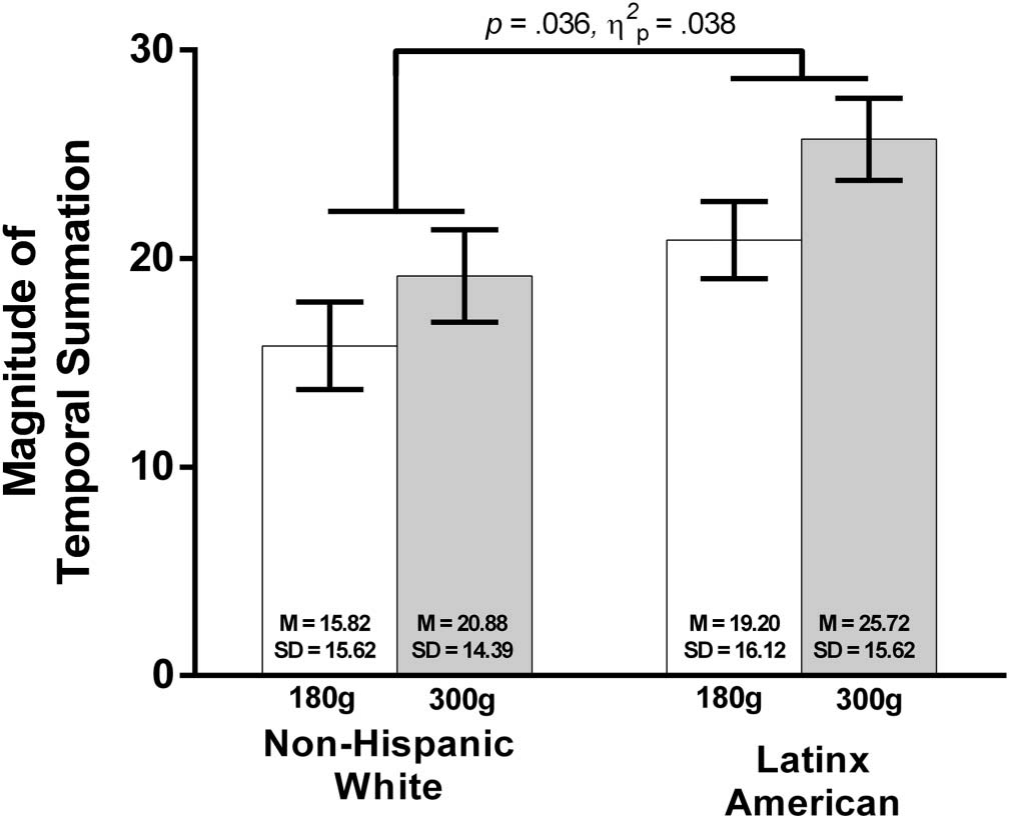
Comparison of differences for mechanical temporal summation to the 180 and 300 g von Frey by ethnicity. Collapsed across body sites and ethnicity, greater summation was demonstrated at 300 g relative to 180 g. Moreover, collapsed across body sites and monofilament weights, Latinx-Americans displayed greater temporal summation relative to NHWs. Mean ± SEM. M_NHW180 g_ = 15.82, SD_NHW180 g_ = 15.62; M_Latinx180 g_ = 20.88, SD_Latinx180 g_ = 14.39; M_NHW300 g_ = 19.20, SD_NHW300 g_ = 16.12; M_Latinx300 g_ = 25.72, SD_Latinx300 g_ = 15.62. NHW, non-Hispanic White.

Figure 2 depicts temporal pain summation between Latinx-Americans and NHWs for the 180 and 300 g weights when averaged across the 3 body testing sites. Analyses revealed significant main effects of monofilament weight, F_1,114_ = 21.29, *P* < 0.001, ${\text{η}}_{p}^{2}$ = 0.157, and ethnicity, F_1,114_ = 4.49, *P* = 0.036, ${\text{η}}_{p}^{2}$ = 0.038, but no weight × ethnicity interaction *(P* = 0.404, ${\text{η}}_{p}^{2}$ = 0.006).

### 3.4. Correlations between adversity and mechanical temporal summation

Temporal summation of mechanical pain significantly correlated across the 3 anatomical sites (metacarpal, phalanx, and trapezius) and 2 filament weights (180 and 300 g) (*r* ≥ 0.56, *P* < 0.001). Therefore, temporal summation of mechanical pain was averaged across the 3 sites to create an average total mechanical temporal summation of pain that was used in subsequent correlation analyses.

Table 2 displays correlations separated by ethnicity. A complete set of correlation matrices separated by ethnicity are included in Supplemental Tables 1 and 2 (available at http://links.lww.com/PR9/A73 and http://links.lww.com/PR9/A74). The trauma subscale, composite, and symptom scores were not significantly associated with temporal summation for either ethnicity group. Recent and lifetime experiences of ethnic discrimination were both inversely associated with mechanical temporal summation, but only for Latinx-Americans. However, there were no significant differences between the discrimination–temporal summation correlations across ethnicities (Table 3).

**Table 2:** Ethnicity correlations comparisons between temporal summation demographics, mood, trauma, discrimination, and social status.

Variables	Ethnicity	
	Non-Hispanic White	Latinx-American
Demographic		
Gender	0.06	−0.03
Mood		
Depressive symptoms (CESD)	0.10	−0.01
Perceived stress (PSS)	−0.08	0.05
Trauma		
Trauma total (ETISR)	−0.09	−0.17
Trauma general (ETISR)	0.00	−0.05
Trauma physical (ETISR)	−0.11	−0.06
Trauma emotional (ETISR)	−0.16	−0.17
Trauma sexual (ETISR)	0.15	−0.14
Trauma—disturbance symptoms (ETISR)	0.04	−0.24
Trauma—dissociative symptoms (ETISR)	−0.01	0.07
Discrimination		
Ethnic discrimination—recent (GEDS)	−0.14	−0.32†
Ethnic discrimination—lifetime (GEDS)	−0.24	−0.34†
Ethnic discrimination—appraisal (GEDS)	−0.11	−0.22
Objective social status		
Father's education	0.03	0.22
Mother's education	−0.03	0.22
Household income—current	−0.22	0.02
Household income—childhood	0.09	0.10
Δ objective social status	−0.22	−0.39†
Subjective social status		
Subjective social status—current	0.07	−0.03
Subjective social status—childhood	0.23	0.23
Δ subjective social status	−0.15	−0.30*

Gender coded 0 = women, 1 = men; income coded 1 = less than $5,000, 2 = $5,000 through $11,999, 3 = $12,000 through $15,999, 4 = $16,000 through $24,999, 5 = $25,000 through $34,999, 6 = $35,000 through $49,9997 = $50,000 through $74,999, 8 = $75,000 through $99,999, 9 = $100,000 and greater; Father and Mother Education Coded 1 = elementary school or less, 2 = middle school, 3 = some high school, 4 = high school graduate/GED equivalent, 5 = postsecondary school other than college, 6 = some college, 7 = college graduate, 8 = some graduate school, 9 = gaduate degree; CES-D = Center for Epidemiological Studies Depression Scale; ETISR, Early Traumatic Inventory Self-Report; GEDS = General Ethnic Discrimination Scale; PSS = Perceived Stress Scale; Δ objective social status = current household income − childhood household income; Δ subjective social status = current subjective social status − childhood subjective social status.

* *P* < .05.

† *P* < .01.

**Table 3:** Difference between correlation coefficients across ethnicity.

	Non-Hispanic White			Latinx-American			*z*	*P*
	N	*r*	*P*	N	*r*	*P*		
Ethnic discrimination (GEDS)								
Recent	51	−0.138	0.333	65	−0.322	0.009	−0.99	0.322
Lifetime	51	−0.237	0.094	65	−0.338	0.006	0.57	0.569
Social status								
Δ objective social status	41	−0.22	0.167	52	−0.394	0.004	0.89	0.374
Δ subjective social status	51	−0.152	0.287	64	−0.298	0.017	0.80	0.424

None of the cross-sectional childhood or present-day markers of objective social status or subjective social status were significantly associated with mechanical temporal summation for Latinx-Americans or NHWs. Changes in objective (ie, family household income) and subjective social status across the lifespan were significantly and inversely associated with mechanical temporal summation for Latinx-Americans, but not for NHWs. However, there were no significant differences between Δ social status–temporal summation correlations across ethnicities (Table 3).

## 4. Discussion

The current study found that Latinx-Americans reported experiencing significantly greater trauma, greater discrimination, and lower social status. Latinx-Americans likewise experienced significantly greater temporal summation. Finally, increased recent and lifetime experiences of ethnic discrimination, along with upward changes in objective and subjective social status across the lifespan, significantly correlated with decreased temporal summation for Latinx-Americans (Table 2). However, there were no significant differences between the correlation coefficients for Latinx and NHWs (Table 3).

Greater temporal summation for Latinx-Americans seen in the current study is consistent with a recent study examining laboratory pain sensitivity in a large combined cohort of participants identifying as either healthy or having temporomandibular disorder across ethnicity groups.^60^ Considering evidence supporting the clinical relevance of dynamic laboratory pain sensitivity measures,^24,26,38,83^ greater summation may represent a potential pain risk factor for Latinx-Americans.‬‬‬‬‬‬‬‬‬‬‬‬‬‬‬‬‬

### 4.1. The relationship between ethnicity, adversities, adversity correlates, and mechanical temporal summation of pain

Consistent with prior literature,^72^ Latinx-Americans in the current study reported greater trauma experiences relative to NHWs. Trauma experiences did not significantly correlate with greater temporal summation for either ethnic group. Contrary to the current study's hypotheses, greater recent and lifetime ethnic discrimination experiences significantly correlated with lower temporal summation for Latinx-Americans. The negative relationship between discrimination responses and temporal summation, however, was comparable to the NHW group.

The current findings differ from previous studies that observe that greater childhood adversity and ethnic discrimination associated significantly with enhanced central sensitization markers.^54,90,91,93^ It is conceivable that the differences in patterns between the studies may be due to differences in the samples studied. For example, although some previous studies examining childhood trauma and central sensitization markers also sampled pain-free young adults within a university setting, such studies actively recruited samples stratified on childhood adversity, consisting predominantly of NHWs.^90,91,93^ Regarding racial discrimination, earlier laboratory studies focused only on discrimination and pain sensitivity among African Americans and associations with central sensitization markers have been inconsistent. For example, a significantly positive relationship between discrimination and temporal summation occurred in one study consisting primarily of African Americans with chronic pain from the community,^54^ but another study observed no significant relationship for pain-free African Americans.^12^ Even beyond laboratory pain testing studies, recent evidence suggests that perceived ethnic discrimination correlates significantly with greater clinical pain intensity (*r* = 0.21) and pain disability (*r* = 0.27) among Latinx individuals recruited from a government-subsidized community-based outpatient clinic.^6^ However, Bakhshaie et al.'s (2019) study composed largely of non-U.S.-native participants (88.4%) who predominantly spoke Spanish as their first language (96.6%) and earned less than $14,999 per year (55.9%),^6^ a contrast to the U.S.-native Latinx-Americans from higher household incomes assessed in the current study (Table 1). The stated hypotheses in the current study regarding the relationship between trauma and discrimination was derived from the aforementioned laboratory pain testing studies that assessed either a different ethnicity group (African Americans), a different trauma group (ie, stratified high vs low trauma), or a different setting (ie, community population). Although African Americans and Latinx-Americans are both minoritized people of color, there are fundamental differences between the 2 populations including generational trauma experiences and discrimination (eg, history of slavery and current political climate around U.S. immigration). Recent work would also suggest that heterogeneity within a minoritized population can also contribute to differences in clinical pain outcomes.^6^ However, considering expected Latinx-American growth over decades in the United States, along with the unique histories and current sociopolitical realities experienced by Latinx-Americans and their subgroups (eg, Mexican, Central-American, South-American), it is important to evaluate the specific contexts (eg, regions, study settings, and socioeconomic status), cultures (eg, values, beliefs, histories, and nativity), and adversities (eg, trauma and discrimination) that may contribute to their pain experiences. Specifically, future studies must resist simple extrapolation from other groups.

Of the status markers, neither objective nor subjective social status indices during childhood or present day significantly related to temporal summation across groups. This finding differs from prior work describing significant relationships between social status and physical pain^8,28,30,32,40,44,48,57,62,66,81,87^ and temporal summation.^38^ Instead, downward changes in objective (ie, family household income decreased over time) and subjective social status (ie, subjective social status decreased over time) across the lifespan—such that individuals reported a decrease in status since childhood—significantly correlated with greater temporal summation for Latinx-Americans. However, there were no significant differences between ethnicity groups for either correlation coefficient. The relationship between downward social status across the lifespan and pain in the current study is consistent with prior work demonstrating significant relationships with downward objective social status across the lifespan and low back pain in adulthood.^48,52^ The current study extends the literature by demonstrating that both downward objective and subjective social status across the lifespan correlates with enhanced temporal summation, a central sensitization proxy. In line with social causation hypotheses,^22^ downward social status may put individuals at future risk for chronic pain by promoting central sensitization, whereas maintained or increased status may reduce risk. Given evidence suggesting pain conditions, such as chronic back pain, relate with greater temporal summation,^56,80^ the current findings may offer insight into the relationship between social status across the lifespan, central sensitization, and chronic pain risk.

### 4.2. Limitations

The current study possesses limitations for consideration. First, this study used cross-sectional data with correlations, limiting causal interpretations and suggesting the need for future longitudinal research.

Second, this study tested pain-free individuals to examine whether ethnicity and adverse experiences related to endogenous pain processing phenomenon that could contribute to chronic pain risk. Although temporal summation is greater in individuals with clinical pain,^65,78^ enhanced temporal summation of pain also occurs in nonclinical populations,^13,55^ suggesting that the risk for greater pain severity may manifest before clinical pain development. However, although studying a pain-free sample allows for examining group differences and relationships while ruling out the influence of disease status, it also limits the ability to know whether the findings generalize to Latinx-Americans experiencing clinical pain.

Third, university samples limit the current study to presumably more resilient, high-functioning individuals of greater socioeconomic status who may have experienced less traumatic and discriminatory events and had greater resources to cope. Although the sample consists of U.S. natives, excluding any first-generation immigrants who may experience greater adversities, demographic research suggests 27% to 47% of second-and-higher generation Latinx-Americans have experienced recent maltreatment because of their ethnicity.^51^ Nevertheless, considering consistent relationships between pain, trauma,^3,9,42,43,74,79,82,90,91,92–93^ and socioeconomic status,^8,28,30,32,40,44,48,57,62,81,87^ the current study's makeup may too restricted to observe intersectional interactions between ethnicity, adversity, and temporal summation. Thus, future replication of the current study among more diverse community and nonnative samples with lower socioeconomic status is warranted.

Fourth, a male, African-American experimenter conducted all laboratory procedures. His demographics could have affected participants' responses even with efforts to minimize observer bias.

Fifth, the current study's ETISR measure did not assess trauma past 18 years of age. Childhood trauma was of primary interest, given Latinx-Americans disproportionately experience childhood trauma relative to NHWs.^50,72^ Therefore, future studies should evaluate lifetime trauma experiences.

Finally, although we randomized laboratory task order before temporal summation testing, equated the same procedures before the temporal summation task across participants, and enforced rests between tasks, participants in the current study completed several tasks. Carryover effects are possible, potentially moderating the current results. Therefore, the current study warrants replication.

### 4.3. Conclusions

The current study is the first to observe greater mechanical temporal summation of pain among completely pain-free Latinx-Americans relative to NHWs. Although Latinx-Americans in the current study also reported greater adversities and adversity correlates, counter to hypotheses and earlier work among African Americans, greater recent and lifetime experiences of ethnic discrimination associated with *less* temporal summation, suggesting that different mechanisms may underlie the relationship between discrimination and pain for Latinx-Americans and African Americans. However, future research will need to inspect discrimination measures more critically to make sure standard self-report measures are equally valid, representative of the most important discrimination events, and predictive of different pain outcomes among unique minoritized demographic groups.

In a novel finding, decreases in objective and subjective social status across the lifespan (from childhood to the present) correlated with greater temporal summation. Dynamic social status measures that reflect change across the lifespan may predict centralized pain risk above solely assessing current or cross-sectional socioeconomic status measures. Future research is needed to explore whether this relationship is not only replicable, but also incrementally predictive for pain risk among community samples.

Taken together, the present findings highlight the complex association between adversities, adversity correlates, and pain. A better understanding of the impact of such experiences for this particularly important, yet understudied, group may help explain the mechanisms underlying the relationship between adversities, adversity correlates, and pain risk for Latinx-Americans.

## Disclosures

The authors have no conflicts of interest to declare.

## Appendix A. Supplemental digital content

Supplemental digital content associated with this article can be found online at http://links.lww.com/PR9/A73 and http://links.lww.com/PR9/A74.
